# Supplementary material for: Time-series transcriptome analysis identified differentially expressed genes in broiler chicken infected with mixed Eimeria species
Source: Front Genet. 2022 Aug 8;13:886781. doi: 10.3389/fgene.2022.886781 (PMC9393255; doi:10.3389/fgene.2022.886781)
Supplement: Supplementary file 2 [file DataSheet1.ZIP › 4dpi_KEGG.Gsea.1625100298584/KEGG_VASCULAR_SMOOTH_MUSCLE_CONTRACTION.html]

Details for gene set KEGG\_VASCULAR\_SMOOTH\_MUSCLE\_CONTRACTION[GSEA]

|  || Dataset | TMM\_4dpi\_gct\_format\_4dpi\_gct\_format.Class\_4dpi.cls #PC\_versus\_NC.Class\_4dpi.cls #PC\_versus\_NC\_repos |
| Phenotype | Class\_4dpi.cls#PC\_versus\_NC\_repos |
| Upregulated in class | 1 |
| GeneSet | KEGG\_VASCULAR\_SMOOTH\_MUSCLE\_CONTRACTION |
| Enrichment Score (ES) | 0.36892426 |
| Normalized Enrichment Score (NES) | 1.3760055 |
| Nominal p-value | 0.046580773 |
| FDR q-value | 0.4656887 |
| FWER p-Value | 0.9996 |
Table: GSEA Results Summary

  

Fig 1: Enrichment plot: KEGG\_VASCULAR\_SMOOTH\_MUSCLE\_CONTRACTION      
 Profile of the Running ES Score & Positions of GeneSet Members on the Rank Ordered List

  

| SYMBOL | TITLE | RANK IN GENE LIST | RANK METRIC SCORE | RUNNING ES | CORE ENRICHMENT || 1 | PLA2G12B | na | 115 | 1.564 | 0.0548 | Yes |
| 2 | CACNA1D | na | 447 | 1.019 | 0.0691 | Yes |
| 3 | PLA2G6 | na | 448 | 1.018 | 0.1111 | Yes |
| 4 | MYLK3 | na | 569 | 0.901 | 0.1382 | Yes |
| 5 | PRKCD | na | 605 | 0.875 | 0.1713 | Yes |
| 6 | ADCY8 | na | 750 | 0.789 | 0.1918 | Yes |
| 7 | RAMP3 | na | 764 | 0.777 | 0.2228 | Yes |
| 8 | PRKCA | na | 853 | 0.736 | 0.2457 | Yes |
| 9 | KCNMB4 | na | 1036 | 0.659 | 0.2577 | Yes |
| 10 | PLCB1 | na | 1082 | 0.644 | 0.2805 | Yes |
| 11 | ADCY7 | na | 1147 | 0.620 | 0.3007 | Yes |
| 12 | CALM2 | na | 1272 | 0.583 | 0.3143 | Yes |
| 13 | ITPR1 | na | 1663 | 0.481 | 0.3015 | Yes |
| 14 | PRKCE | na | 1693 | 0.476 | 0.3187 | Yes |
| 15 | ROCK2 | na | 1922 | 0.436 | 0.3176 | Yes |
| 16 | ADRA1B | na | 2261 | 0.380 | 0.3049 | Yes |
| 17 | PRKX | na | 2262 | 0.380 | 0.3206 | Yes |
| 18 | ARHGEF12 | na | 2265 | 0.380 | 0.3361 | Yes |
| 19 | PPP1CB | na | 2368 | 0.367 | 0.3427 | Yes |
| 20 | RHOA | na | 2375 | 0.366 | 0.3573 | Yes |
| 21 | MYL6 | na | 2730 | 0.318 | 0.3407 | Yes |
| 22 | ADORA2B | na | 2861 | 0.301 | 0.3422 | Yes |
| 23 | ADORA2A | na | 2956 | 0.288 | 0.3462 | Yes |
| 24 | MYLK | na | 2963 | 0.287 | 0.3576 | Yes |
| 25 | ACTG2 | na | 3279 | 0.246 | 0.3413 | Yes |
| 26 | CALD1 | na | 3321 | 0.241 | 0.3478 | Yes |
| 27 | ROCK1 | na | 3380 | 0.235 | 0.3526 | Yes |
| 28 | GNA13 | na | 3499 | 0.221 | 0.3518 | Yes |
| 29 | CALCRL | na | 3556 | 0.213 | 0.3559 | Yes |
| 30 | RAF1 | na | 3562 | 0.212 | 0.3642 | Yes |
| 31 | ADCY2 | na | 3608 | 0.206 | 0.3689 | Yes |
| 32 | AGTR1 | na | 3755 | 0.192 | 0.3646 | No |
| 33 | KCNMB2 | na | 4036 | 0.164 | 0.3479 | No |
| 34 | ARHGEF11 | na | 4243 | 0.147 | 0.3367 | No |
| 35 | EDNRA | na | 4308 | 0.141 | 0.3372 | No |
| 36 | MAP2K2 | na | 4498 | 0.124 | 0.3265 | No |
| 37 | MAPK3 | na | 4570 | 0.119 | 0.3254 | No |
| 38 | MAP2K1 | na | 4692 | 0.107 | 0.3197 | No |
| 39 | ITPR3 | na | 4698 | 0.106 | 0.3237 | No |
| 40 | GUCY1A2 | na | 5137 | 0.066 | 0.2897 | No |
| 41 | PLCB4 | na | 5226 | 0.059 | 0.2848 | No |
| 42 | PRKG1 | na | 5828 | 0.007 | 0.2347 | No |
| 43 | GNAS | na | 5856 | 0.005 | 0.2327 | No |
| 44 | PPP1CC | na | 5925 | -0.001 | 0.2270 | No |
| 45 | CALM1 | na | 6078 | -0.013 | 0.2148 | No |
| 46 | RAMP2 | na | 6173 | -0.020 | 0.2078 | No |
| 47 | PRKCH | na | 6364 | -0.035 | 0.1933 | No |
| 48 | ITPR2 | na | 6378 | -0.037 | 0.1937 | No |
| 49 | ADCY3 | na | 6636 | -0.057 | 0.1746 | No |
| 50 | PLCB2 | na | 6817 | -0.072 | 0.1624 | No |
| 51 | CACNA1C | na | 6906 | -0.080 | 0.1584 | No |
| 52 | PRKACB | na | 7013 | -0.090 | 0.1532 | No |
| 53 | GNA11 | na | 7158 | -0.104 | 0.1454 | No |
| 54 | PPP1R12A | na | 7221 | -0.111 | 0.1448 | No |
| 55 | ACTA2 | na | 7346 | -0.122 | 0.1395 | No |
| 56 | PLA2G4A | na | 7388 | -0.126 | 0.1412 | No |
| 57 | AVPR1A | na | 7446 | -0.131 | 0.1418 | No |
| 58 | PPP1R12B | na | 7672 | -0.150 | 0.1291 | No |
| 59 | MYL9 | na | 7870 | -0.168 | 0.1196 | No |
| 60 | RAMP1 | na | 7958 | -0.175 | 0.1195 | No |
| 61 | PPP1CA | na | 7964 | -0.175 | 0.1263 | No |
| 62 | GNAQ | na | 8151 | -0.193 | 0.1186 | No |
| 63 | ADCY6 | na | 8259 | -0.205 | 0.1181 | No |
| 64 | ARHGEF1 | na | 8511 | -0.230 | 0.1066 | No |
| 65 | NPR2 | na | 8572 | -0.237 | 0.1113 | No |
| 66 | KCNMB1 | na | 8756 | -0.256 | 0.1066 | No |
| 67 | CYP4A22 | na | 8776 | -0.259 | 0.1157 | No |
| 68 | PLA2G4B | na | 8878 | -0.271 | 0.1184 | No |
| 69 | ADCY9 | na | 8880 | -0.271 | 0.1294 | No |
| 70 | BRAF | na | 9677 | -0.371 | 0.0781 | No |
| 71 | MYH11 | na | 9968 | -0.409 | 0.0706 | No |
| 72 | PRKCB | na | 10600 | -0.519 | 0.0391 | No |
| 73 | KCNMA1 | na | 10814 | -0.564 | 0.0445 | No |
| 74 | PLA2G2E | na | 11225 | -0.673 | 0.0379 | No |
| 75 | PLA2G5 | na | 11226 | -0.673 | 0.0657 | No |
Table: GSEA details [plain text format]

  

Fig 2: KEGG\_VASCULAR\_SMOOTH\_MUSCLE\_CONTRACTION      
 Blue-Pink O' Gram in the Space of the Analyzed GeneSet

  

Fig 3: KEGG\_VASCULAR\_SMOOTH\_MUSCLE\_CONTRACTION: Random ES distribution      
 Gene set null distribution of ES for **KEGG\_VASCULAR\_SMOOTH\_MUSCLE\_CONTRACTION**
